# Lunar Caustic in Burns

**Published:** 1846-07

**Authors:** 


*Lunar Caustic in Burns.*—During the autumn of 1840, while the writer was performing a set of experiments in chemistry before a class, a burn was accidentally received in the following manner. In getting out some phosphorus to light a spirit lamp, a little of it dropped into a box of mineral specimens under the table. Being unable to find it readily it was left in the box and forgotten until it got into a blaze. To save the minerals from injury, I attempted to run my left hand under them and the phosphorus and throw all out together; but instead of this, I ran the index and middle finger directly into the blazing phosphorus. The piece, about an inch long, stuck to my fingers, and before I could plunge them into the pneumatic trough, it completely roasted them from their extremities to the second joints. The pain exceeded in intensity any thing that ever I suffered.

But silver and its compounds being the subject of the lecture, there was fortunately a solution of the nitrate on the table, and as soon as reflection returned I plunged my fingers into the caustic liquid. Relief was prompt, and so great that in a few minutes the lecture was resumed. It was then about three o'clock, P. M.; there was slight pain in the fingers till 9, when I fell asleep and suffered no more from it afterwards. Hard crusts of sphacelus shielded the diseased parts till they were thrown off by reproduced flesh beneath. My fingers are now as perfect as ever in shape and motion, and no one could discover a cicatrix.—*Dr. Gordon in Western Lancet.*

---

Dr. Labat, principal physician to the Shah of Persia, and surgeon-in-chief of his armies, who has recently arrived in Paris, attained his position in Persia by curing the Shah of gout, to which it appears he was much subject. He was appointed successively mirza, bey, and khan, and ranks immediately after the princes of the blood royal.—*Med. Gaz.*

---

The sum of 48,000 francs has been demanded from the government by the dean of the Parisian faculty of medicine, to construct a ward in the proposed Hôpital des Cliniques, to contain twelve beds, for the use of sick medical and law students. The conseil académique, and the conseil royal de l'université support the demand.—*Ib.*

---

The cholera, it appears, was introduced into Persia, by the caravans of Steret into the Khorasan, and has passed thence southward to the Kerman, and onwards to the marshy plains of Messendheran and Ghilon, where its ravages were very extensive. At Mesched one-third of the population has perished ; and the population of entire quarters has disappeared at Teheran and Ispahan.—*Ib.*

